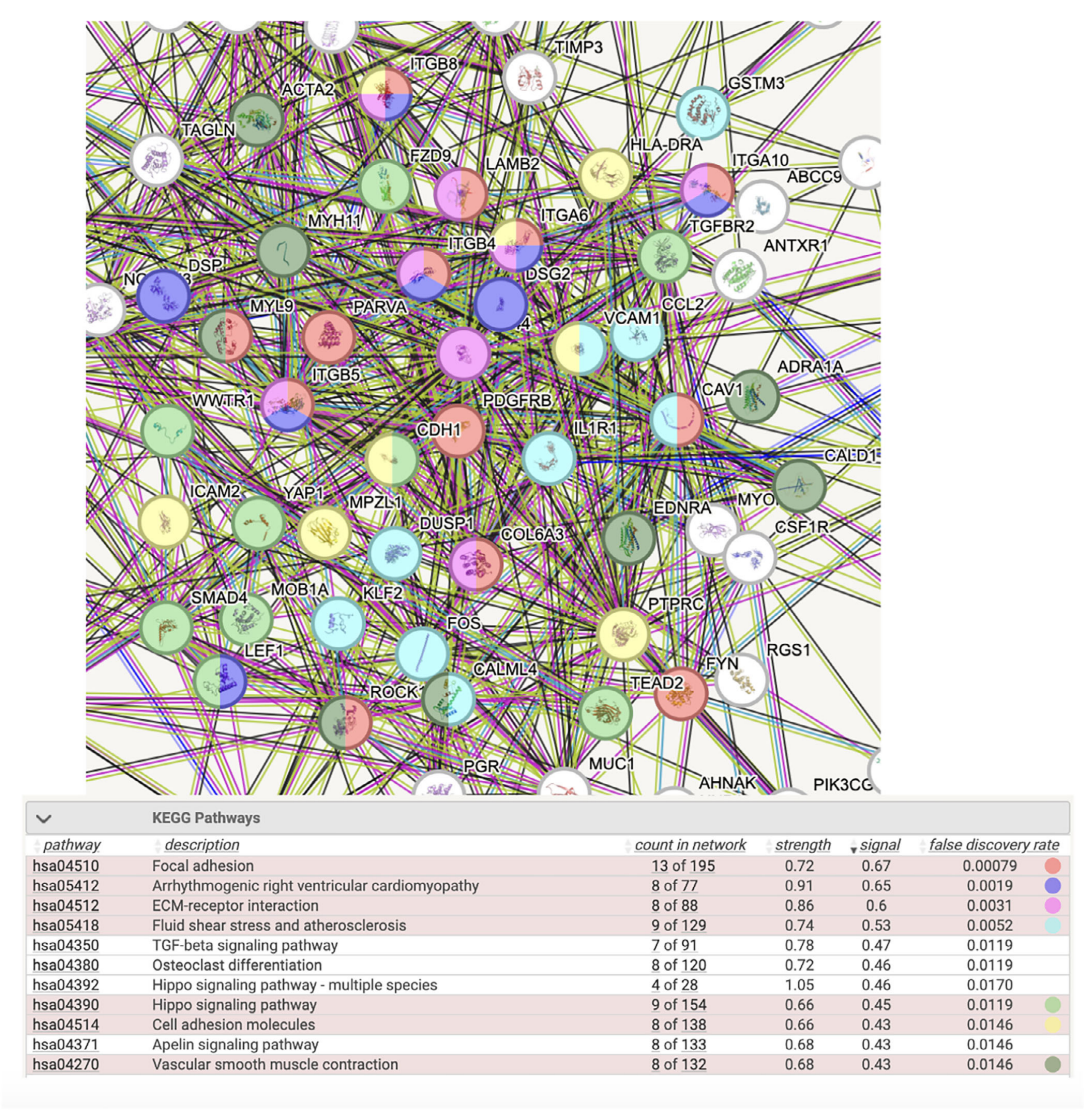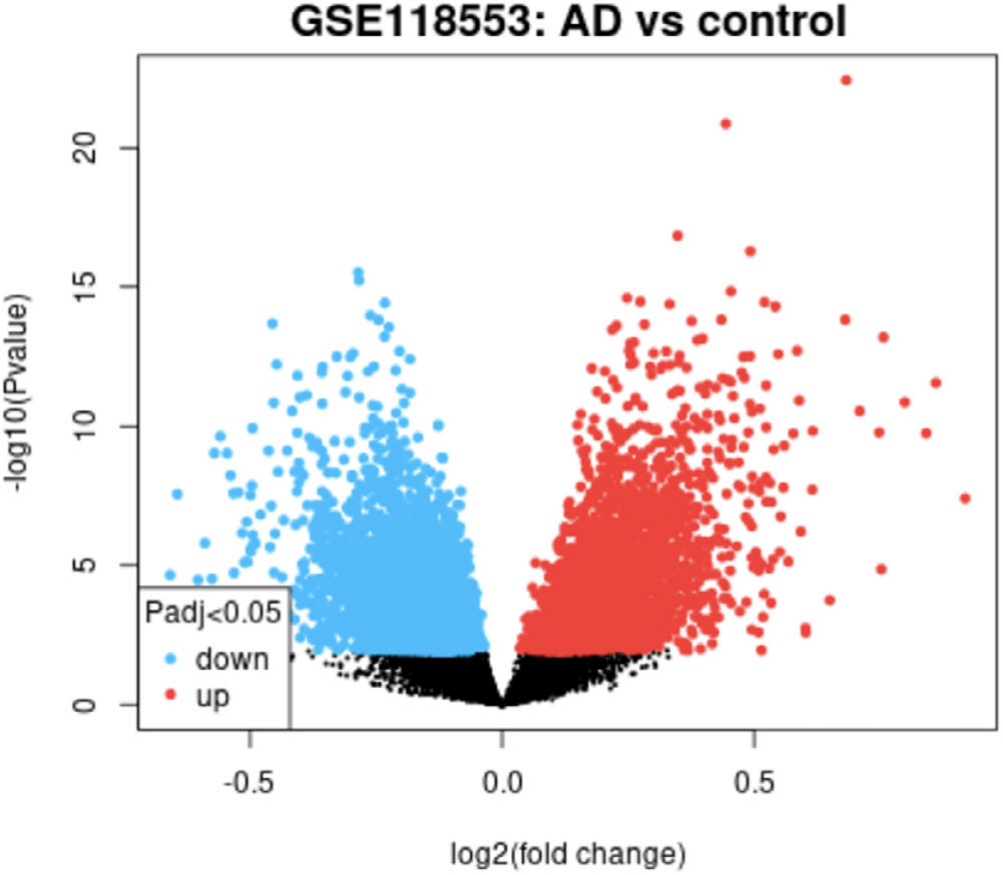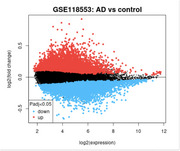# Cellular and Arrhythmogenic Ventricular Cardiomyopathy Pathway in Alzheimer's Disease: Implications for Cardiovascular and Cellular Dysfunction

**DOI:** 10.1002/alz70855_102397

**Published:** 2025-12-23

**Authors:** Meher Garg, Inhan Lee

**Affiliations:** ^1^ SIU School of Medicine, Physican Pipeline Program, Springfield, MI, USA; ^2^ MirCore, Ann Arbor, MI, USA; ^3^ miRcore, Ann Arbor, MI, USA

## Abstract

**Background:**

Alzheimer's Disease (AD) is primarily recognized for its impact on cognition and memory. Emerging evidence highlights its effects on vascular and cellular processes. Dysregulation of cell signaling and cardiovascular pathways may contribute to the progression of the disease and its associated comorbidities. While research has primarily focused on central nervous system degeneration, there is increasing recognition of cardiovascular disease in AD. This study investigates gene expression changes in AD, with a focus on pathways upregulated in the disease that could offer insight into its broader cellular and cardiovascular dysfunction.

**Method:**

Gene Expression Omnibus (GEO) dataset GSE132903 was analyzed to examine RNA expression in the middle temporal gyrus from 52 AD patients and 55 controls. Differential gene expression was assessed using GEO2R, followed by pathway enrichment analysis through STRING‐DB. Key Kyoto Encyclopedia of Genes and Genomes (KEGG) pathways related to cellular processes, cardiovascular function, and cancer were examined. Statistical significance was determined through FDR correction, with results visualized in R/R Studio.

**Result:**

Differential RNA expression analysis revealed significant upregulation of several pathways in AD compared to controls. The Focal Adhesion pathway (hsa04510) was notably upregulated (FDR *p* = 0.00079, strength = 0.67), indicating altered cell‐extracellular matrix interactions. The Arrhythmogenic Right Ventricular Cardiomyopathy pathway (hsa05412) also showed upregulation (FDR *p* = 0.0019, strength = 0.65), suggesting cardiovascular dysfunction. Additionally, pathways related to extracellular matrix (ECM)‐receptor interaction (hsa04512, FDR *p* = 0.0031) and Hippo signaling (hsa04390, FDR *p* = 0.0119) were upregulated, highlighting cellular proliferation and tissue repair mechanisms. Pathways linked to vascular function, such as Vascular Smooth Muscle Contraction (hsa04270, FDR *p* = 0.0146) and Fluid Shear Stress and Atherosclerosis (hsa05418, FDR *p* = 0.0052), were also significantly altered, pointing to potential vascular dysfunction in AD patients

**Conclusion:**

The upregulation of pathways involved in cellular adhesion, cardiovascular function, and tissue repair suggests that AD may disrupt vascular integrity and cellular interactions beyond the central nervous system. These findings provide insight into cardiovascular disease and cardiomyopathy in AD and support the need for further research into these pathways as potential therapeutic targets for managing vascular and cellular dysfunction in AD.